# Gender disparities in end-of-life care: A scoping review of patient, caregiver and care provider perspectives in low-and middle-income countries

**DOI:** 10.1186/s12904-025-01702-9

**Published:** 2025-03-10

**Authors:** Divya Narayanan, Akshaya Sudha Chandrasekaran, Elstin Anbu Raj S, Navya Vyas

**Affiliations:** 1 Department of Global Public Health Policy and Governance, Prasanna School of Public Health, Manipal Academy of Higher Education, Manipal, 576 104 Karnataka India; 2 Center for Evidence-Informed Decision Making, Prasanna School of Public Health, Manipal Academy of Higher Education, Manipal, 576 104 Karnataka India

**Keywords:** End-of-life care, Palliative care, Gender disparities, Low-and middle-income countries

## Abstract

**Background:**

The term “end-of-life” care refers to the physical, social, spiritual, and emotional assistance provided to people near the end of their lives. Throughout history, gender has profoundly impacted many of the decisions people make. Studies have shown an increased demand for palliative care worldwide, which varies significantly among low-and middle-income countries. Achieving universal health coverage requires everyone to have access to health care where and when they need it, irrespective of gender. This scoping review aims to map the evidence available on the gender disparities in end-of-life care in low-and middle-income countries, considering the perspectives of patients, caregivers, and care providers.

**Methodology:**

A comprehensive search was done for the review in the following databases: PubMed, Embase, Scopus, Web of Science, ProQuest, and Cumulative Index to Nursing and Allied Health Literature. The World Health Organization’s definition of gender was the inclusion standard. Studies conducted in low-and middle-income countries were included. Only articles published between 2005 and 2024 were retained. Articles on sexual minorities were excluded.

**Results:**

Eight articles were selected for the review and the descriptive analysis was done, followed by a thematic analysis to synthesize the available data into themes. The themes identified based on the patient’s perspectives were end-of-life preferences, challenges, and perceptions towards caregiving. Care provider perspectives included attitude toward euthanasia and perception towards care provision, while caregiver perspectives involved perceptions of caregiving and challenges. Gender disparities were evident across all perspectives, with women being disproportionately affected.

**Conclusion:**

The review identified significant gender disparities in different aspects of end-of-life care. Women often experience a greater caregiving burden and higher risks of emotional, physical, and sexual violence, while men encounter societal stigma in caregiving roles. Addressing these disparities is required to ensure equitable and inclusive EOL care. Further research should be done in this direction to identify the influence of gender on end-of-life care and how it interacts with other factors like culture, religion, socio-economic status, and education to inform policies that promote gender-sensitive end-of-life care.

**Supplementary Information:**

The online version contains supplementary material available at 10.1186/s12904-025-01702-9.

## Background

The term “end-of-life” (EOL) care refers to the physical, social, spiritual, and emotional assistance provided to the patients, which includes palliative, supportive, and hospice care in the time leading up to a person’s death, with a focus on symptom control rather than cure or prolongation of life [1, 2]. Integrating palliative care into the early phases of disease management has been shown to improve patient outcomes and lower healthcare costs [3, 4]. Such an integration requires the active participation of the patient and their caregivers. In August 2000, the United Nations (UN) Committee on Economic Social and Cultural Rights recognized palliative care as a component of the right to life [5]. This acknowledgment points to the fact that end-of-life requirements will always remain relevant.

According to the Global Atlas of Palliative Care at the End of Life, published in 2014 by the World Health Organization (WHO) in collaboration with the Worldwide Palliative Care Alliance, the global estimate of people requiring palliative care at the end of their lives is about 20.4 million out of which about 78% reside in low- and middle-income countries (LMICs) and 22% in high-income countries. These numbers suggest the increasing global demand for palliative care, which varies significantly among LMICs [6]. As LMICs undergo an epidemiological transition, they face a triple burden of disease, including communicable diseases, injuries and non-communicable diseases like cancer. Consequently, the demand for palliative care is staggering [7–10]. However, palliative care services in such countries are still developing, are mostly restricted to urban areas, and are not integrated into national health systems. Moreover, very little evidence is available about primary palliative care services in LMICs [11–14].

Achieving universal health coverage (UHC) for Sustainable Development Goal 3, good health and well-being, requires that everyone have equitable access to health care where and when they need it, irrespective of their gender [15]. Amongst various factors that are to be addressed to achieve universal health coverage, gender is considered a vital one as it is said to influence various aspects of life like an individual’s health-seeking behavior, access to healthcare, and EOL care, shaping needs, experiences, and expectations [16, 17]. Research indicates that higher levels of gender equality are correlated with better health outcomes, suggesting that addressing gender disparities may contribute to improved healthcare access and quality [18].

Amongst numerous obstacles in health care availability and accessibility, studies show that women in LMICs face higher gender inequality and economic hardships compared to economically advanced countries, and this discrimination feeds the gender-based norms in society [19]. Everywhere in the world, women outlive men. However, the gap in life expectancy between women and men is narrowest in LMICs, with evidence suggesting that while 1 in 3300 women dies from maternal cause in high-income countries, the rate is 1 in 41 in low-income countries [20]. Financial accessibility also comes into the equation, disproportionately affecting women, who are generally thought of as natural caregivers and burdened with familial responsibilities [21]. On the other hand, men are affected by the harmful and rigid norms of toxic masculinity, forcing them to be stoic. Research shows that the support received by men and women at EOL care differs [16, 22]. Such differences can be seen in various components of EOL care, such as care provision, care reception, pain management, choice of life-extending intervention, and place of death. Despite these disparities, the role of gender in EOL care in LMIC is widely under-researched. Very minimal evidence is available to substantiate or refute the actual role of gender in the said care. This scoping review aims to map the existing evidence available on the gender disparities in EOL care in LMICs, considering the perspectives of patients, caregivers, and care providers. It will contribute to a better understanding of the areas in EOL care where gender disparities exert influence, which will be crucial in informing future efforts toward more equitable access to EOL care.

## Methodology

### Study design and rationale

A scoping review was done to identify and map the existing literature on gender disparities in EOL care in LMICs. This review follows the Preferred Reporting Items for Systematic Reviews and Meta-analyses extension for Scoping Reviews (PRISMA-SCR) guidelines [23] and Joanna Briggs Institute (JBI) framework [24]. The review protocol was registered on the Open Science Framework [25].

### Eligibility criteria

Inclusion and exclusion criteria were clearly defined at the beginning of the review. The WHO’s definition of gender was taken as the inclusion standard. Studies conducted in LMICs, as classified by the World Bank, were included. Articles published between 2005 and 2024 were included, as this period comprised of major literature in the area of gender and palliative care as observed in PubMed. ProQuest database for thesis and dissertation was considered as grey literature. Articles published in languages other than English were excluded. Studies focusing on sexual minorities were also excluded as they did not align with the purpose of the review. No other specific literature was excluded.

### Search strategy

The search strategy was developed by E.A.R and focused on two concepts– end-of-life care and gender. Terms for these two concepts were combined using the Boolean operators “AND” and “OR.” The following terms were used: “palliative care,” “end of life care” combined with “gender disparity” “gender differences,” and “gender inequality”.

### Data sources

A systematic and comprehensive search was conducted across six major databases: PubMed, Embase, Scopus, Web of Science (WOS), ProQuest, and Cumulative Index to Nursing and Allied Health Literature (CINAHL). This search was conducted over a period of three months, from July to September 2024. These databases were chosen to ensure the retrieval of quality articles.

### Selection of studies

All articles identified in the search were exported to Rayyan a.i [26]., and duplicates were removed. Title and abstract screening were done independently by two reviewers, D.N and A.S.C, with the blind mode enabled to minimize bias, preventing reviewers from seeing each other’s decisions. Later, blind mode was turned off, and conflicts were resolved by N.V. The included articles were then subjected to full-text screening. These retained articles were later subjected to blinded full-text screening by two reviewers, D.N and A.S.C. Based on the eligibility criteria as mentioned above, the articles were selected for review. Finally, cross-referencing of the selected articles was done to identify additional articles for inclusion in the review.

### Data extraction and synthesis

Microsoft Excel (2024) was used to extract the data. Two reviewers independently extracted information on the following: Title, author, year of publication, country details, study type, whether gender differences were identified, number of participants, age of participants, and type of perspectives. The results were presented in the table given below (Table 1) to provide a clear overview of the information collected.

**Table 1:** Data extraction table

Author, Year	Country	Type of Study	Gender difference	No.	Age	Perspective	Results
Qamar Abbas et al., 2008	Pakistan, India	Cross-sectional	Yes	112	Mean age - Pakistan- 42.8 yrs India- 35.4 yrs	Care Provider	**Care Provider perspective: Attitude towards euthanasia** - In Pakistan, male doctors were more likely to be in favor of euthanasia compared to female doctors while no such differences were found in India.
Kamath et al., 2011	India	Cross-sectional	No	213	Not given	Care Provider	**Care Provider perspective: Attitude towards euthanasia** - In India, there was no significant difference in opinion regarding euthanasia between the genders
Dworzanowski-Venter, 2017	South Africa	Qualitative	Yes	18	Not given	Caregiver and Care Provider	**Care Provider perspective: Perception towards care provision–** Female oncology nurses viewed emotional labor as central to their job, while males regarded it as peripheral. Male nurses also faced stigma, including being thought of as “gay” due to their profession. **Caregiver perspective: Perceptions towards caregiving -** Caregiving is seen as “women’s work”, conflicting with traditional norms of masculinity. Male caregivers reported that caregiving requires an emotional component that conflicts with societal expectations of masculinity. They face pressure to pursue a more masculine careers and remain in caregiving only if the professional recognition outweighs societal judgments. Women, seen as natural caregivers, are expected to prioritize other’s needs over their own.
Vijayalakshmi et al., 2018	India	Cross-sectional	Yes	214	Mean age − 36.8 +/- 9.1 yrs	Care Provider	**Care Provider perspective: Attitude towards euthanasia**– Among Indian nurses, males were more supportive of euthanasia, endorsing patient autonomy in decisions like stopping nutritional support if a patient desired euthanasia. Female nurses opposed ceasing CPR in cases of sudden respiratory and cardiac arrest when a patient desires euthanasia and were less likely to view caring for a terminally ill patient as a burden for relatives. No gender differences were found in attitudes regarding euthanasia application, the right to live decently, and family involvement in euthanasia decisions. However, more females disagreed with the statement that “fear of death shows differences due to religious beliefs”.
Jorge et al., 2019	Brazil	Cross-sectional	Yes	400	Mean age − 69yrs	Patient	**Patient perspective: End-of-life preferences -** Men significantly preferred home as place of death than women.
Jorge et al., 2019	Brazil	Cross-sectional	Yes	400	Mean age − 69yrs	Patient	**Patient perspective: End-of-life preferences -** Preference to always be informed about symptoms and problems was greater among men than women. Being a woman was found to be a significant protective factor for always wanting to be informed about limited time left.
Mkandawire-Valhmu et al., 2020	Malawi	Qualitative	Yes	40	Patient age- 18–24 (1), 25–34 (6), 35–44 (4), 45–64 (11), 65+ (1), unknown − 3 Caregiver age- 18–29 (3), 30–49 (4), 50–59 (7)	Caregiver and patient	**Patient perspective: Challenges -** Female patients at EOL faced emotional, physical, and sexual violence, with many abandoned by partners when too weak for intimacy. **Caregiver perspective: Perceptions towards caregiving–** Caregiving responsibilities often fall on females, even minors, impacting their education despite the presence of male household members. **Caregiver perspective: Challenges -** Women caregivers reported increased domestic violence, including physical, emotional, and sexual abuse during EOL caregiving, when their coping and problem-solving capacities were strained. Many women caregivers viewed marriage as financial security. However, this expectation often collapsed during EOL caregiving, as some men abandoned their spouses or escalated acts of violence, further compromising their caregiving quality.
Shahbaz et al., 2022	Pakistan	Cross-sectional	Yes	60	18–85 yrs	Patient	**Patient perspective: Perception towards caregiving -** Both male and female patients perceived females as more quality caretakers due to their warmth, politeness, care, and love. However, female cancer patients were found to respond more positively to male caregivers, attributing this to their empathy.

LEGEND: AUTHOR, YEAR: Primary author(s) of the study and the year of publication; COUNTRY: Country or countries where the study was conducted; TYPE OF STUDY: Research design used in the study (e.g., Cross-sectional, Qualitative); Gender Difference (Yes/No): Indicates whether gender differences were analyzed in the study; No.: Total number of participants included in the study; Age: Age distribution or mean age of participants; Perspective: Perspective(s) represented in the study, such as Care Provider, Caregiver, or Patient; Results: Results identified

## Results

### Study characteristics

**Fig. 1:**
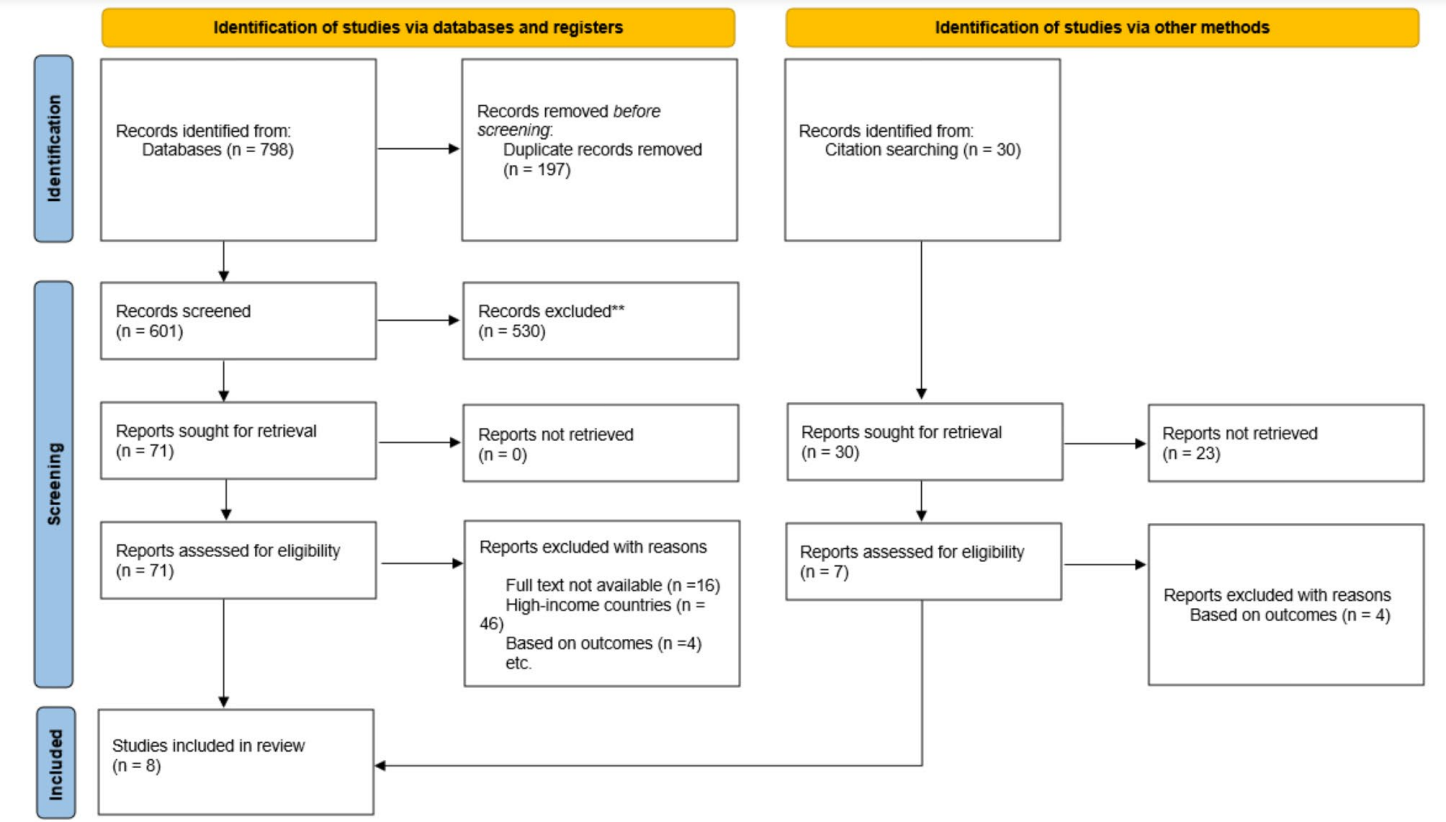
PRISMA flow diagram

A total of 798 articles were retrieved during the systematic search (Fig. 1). After the elimination of 197 duplicates, 601 articles were taken up for title and abstract screening. During this process, two reviewers independently screened the articles based on the inclusion criteria. At the end of this process, 71 articles were deemed fit for full-text screening. After the full-text screening by two reviewers, 16 articles were removed due to the unavailability of full text, 46 articles were excluded because the study was conducted in high-income countries, and four articles were removed as they did not discuss about gender differences. This left a total of five articles in the review (Fig. 1). Additionally, by looking through the included papers’ references, three articles that satisfied the inclusion criterion were found. In total, eight articles were selected for the scoping review.

The total number of articles selected for review after full-text screening was eight. Out of these, seven articles highlighted gender differences, while one did not. Cross-sectional quantitative study designs were the most common, comprising six out of the eight studies [27–32], while the rest were qualitative [33, 34]. The studies were conducted in the following countries: India– 2 [27, 29], Brazil − 2 [30, 31], South Africa − 1 [33], Malawi − 1 [34], Pakistan– 1 [32] and one study covered both India and Pakistan [28]. Out of the eight articles retained for review, four articles analyzed the attitudes of medical professionals towards EOL care, four focused on patient perspectives, and two examined caregiver perspectives. Cancer was the most common disease associated with EOL care, discussed in four articles.

### Gender perspectives

**Fig. 2:**
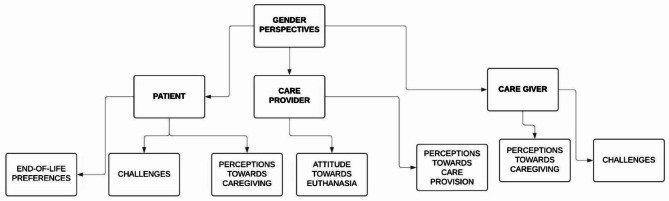
Themes identified based on perspectives

All the studies selected for the review underwent descriptive analysis, and the findings are presented in Table 1. This was followed by a thematic analysis to synthesize the available data into themes. The themes identified were grouped according to the perspectives of the patients, caregivers, and care providers (Fig. 2).

#### Patient perspective [30–32, 34]

1. End-of-life preferences: Male patients exhibited a stronger preference for dying at home and to always be informed about their prognosis and remaining time compared to female patients.
2. Challenges: Female patients at EOL faced heightened vulnerability to emotional, physical, and sexual violence, exacerbating their distress. Social stability associated with marriage often crumbled, as many women were abandoned by their partners when they became too weak to satisfy them sexually.
3. Perception towards caregiving: Both male and female patients perceive females as more nurturing and quality caretakers than males. However, an interesting contrast emerged among female cancer patients, who tended to respond more positively to male caregivers, which was attributed to the empathy male caretakers showed towards the patients.

#### Care provider perspective [27–29, 33]

1. Attitude towards euthanasia: Gendered perspectives on euthanasia varied across cultural contexts. Male healthcare providers were generally more supportive of euthanasia-related decisions than their female counterparts, believing that patients should have the right to decide. Religious beliefs significantly influenced attitudes toward fear of death, with female healthcare providers being less likely than their male counterparts to believe that fear of death varies based on religious beliefs.
2. Perception towards care provision: Among oncology nurses, females viewed emotional labor as central to their job, while their male counterparts regarded it as a more peripheral aspect of their job. Male nurses also struggled with the stigma associated with caregiving as a gender-disruptive career choice, sometimes leading to societal perceptions that questioned their masculinity.

#### Caregiver perspective [33, 34]

1. Perceptions towards caregiving: Caregiving was predominantly seen as “women’s work,” reinforcing traditional gender roles. Male caregivers often struggled with societal expectations, feeling that caregiving required emotional sensitivity, which conflicted with the ideas of masculinity. Their involvement was only accepted when it was accompanied by professional recognition that outweighed societal judgments. Meanwhile, female caregivers were expected to prioritize caregiving, often at a personal cost, with caregiving responsibilities falling on minors, even in households with adult men.
2. Challenges: Women caregivers faced multiple vulnerabilities, including increased exposure to domestic violence. The pressures of caregiving amplify power balances, with some women experiencing physical, emotional, and sexual abuse during EOL caregiving. Many women felt that economic constraints further made caregiving difficult and sought marriage as a solution, which often failed to provide the security they wanted.

## Discussion

The review’s aim was to synthesize and consolidate the existing knowledge about the impact of gender on EOL care in LMICs. The review assessed the existing gender differences in EOL through the lens of care providers, caregivers, and patients. The findings highlight the complex interplay between gender, societal norms, caregiving roles, and patient preferences at the EOL. The review provides a more nuanced understanding of how traditional gender norms involuntarily position women as primary caregivers, even when they are underage [32, 34]. On the other hand, males are discouraged and stigmatized from taking up caregiving responsibilities despite their willingness because of the societal pressure to confront their stoicism [33]. Males and females were viewed from opposing angles by society, either forcing or refraining them from caregiving [16].

Similarly, gender disparities emerged in patient preferences, with men more likely to prefer home as the place of death and to seek detailed prognostic information [30, 31]. Although most of the patients consider females to be quality caregivers due to their emotional availability and feminine warmth [33, 34], there were still certain female patients who thought male caregivers had better empathizing capacity [32], which went against the status quo.

The findings also reveal how socio-economic factors intersect with gender to shape caregiving dynamics and patient experiences. For instance, in EOL care, many women, caregivers, and patients alike face additional vulnerabilities such as domestic violence, abandonment, and resource constraints, which adversely affect the quality of care and life [34].

The emphasis on euthanasia among care providers, in contrast to patients and caregivers, may stem from their role in navigating the ethical and medical complexities of EOL care. Providers often face moral distress when managing prolonged suffering, which may explain their focus on euthanasia as a potential solution. Another key finding was that female care providers considered emotions as central to their work and were opposed to certain euthanasia-related decisions, such as stopping nutritional support and life-extending interventions during emergencies [28, 29, 33]. This may be attributed to females being more sensitive in matters of life and death, while their male counterparts go for a logical approach by being in favor of euthanasia, believing that the patient’s decision should be respected as caring for a terminally ill patient was a burden for relatives [29, 33]. Despite these differences, no gender differences were observed in attitudes regarding euthanasia application, family involvement in euthanasia decisions, and the right to live decently [27].

Building on the findings discussed above, it is evident that gender disparities exist in all spheres of end-of-life care. These disparities are rooted in societal gender norms, which are not just external but also internalized by individuals, further influencing our professional choices, personal decisions, and life trajectories.

The study had a few limitations. The definition of gender used in this review is a heteronormative one, which might be considered rigid and less inclusive of the non-binary concept of “gender identity.” While the understanding of gender identity is still evolving, that area requires a more structured approach. By focusing on binary gender in this review, we aim to provide a foundation that can act as a stepping stone for future studies exploring the intersection of gender identity and EOL care. Another limitation is that generalizing the findings to all LMICs poses a challenge due to the limited number of research articles addressing the gender aspect in EOL care. The current review identified numerous studies centered on EOL care, but the effect of gender remained largely unexplored, particularly in LMICs. This is further influenced by the relatively lesser number of research being conducted in LMICs.

Further research is required to gain a deeper understanding of the interplay of gender with other critical factors such as culture, religion, socio-economic status, and education as these elements contribute and shape the variations on gendered expectations. Investigating such intersectionality is important to uncover the complex ways in which they influence EOL care perceptions and practices. Culturally competent, financially accessible and gender-sensitive EOL care frameworks can address disparities for all individuals. In the longer run, this research will aid the formulation of gender-inclusive policies, ensuring that the EOL care needs of all individuals are addressed, irrespective of their gender.

## Conclusion

Overall, this review highlights significant gender disparities in all aspects of EOL care in LMICs, with the disparities being more prevalent among women, particularly in their roles as caregivers and patients. However, the extent to which gender, in combination with other factors like culture, religion, socio-economic status, and education, influences said care needs to be further explored. EOL care remains largely under-researched and warrants more active investigation. Future research in this domain should take a more inclusive approach, focusing on all aspects of EOL care, including the experiences of non-binary and transgender individuals, to better understand their unique needs. Additionally, given that this review included studies from a limited number of LMICs, the generalizability of the findings should be considered. Expanding the geographical scope in future studies could provide a more comprehensive understanding of gender disparities in EOL care, which can prove pivotal for achieving health equity and better health outcomes.

## Electronic supplementary material

Below is the link to the electronic supplementary material.

Supplementary Material 1

Supplementary Material 2

## Data Availability

No datasets were generated or analysed during the current study.

## Declarations

### Data and material availability

Since no datasets were created during the review process, data sharing is not applicable to this article.

## Abbreviations

EOL: End of Life
LMIC: Low-and Middle-Income Countries
PRISMA-SCR: Preferred reporting items for systematic reviews and meta-analyses- Scoping Review
JBI: Joanna Briggs Institute
CPR: Cardiopulmonary Resuscitation
