# Supplementary material for: Gender disparities in end-of-life care: A scoping review of patient, caregiver and care provider perspectives in low-and middle-income countries
Source: BMC Palliat Care. 2025 Mar 10;24:62. doi: 10.1186/s12904-025-01702-9 (PMC11892201; doi:10.1186/s12904-025-01702-9)
Supplement: Supplementary file 1 — Supplementary Material 1 [file 12904_2025_1702_MOESM1_ESM.pdf]

S.no	Database	Search Strategy	Hits
1.	PubMed	("gender differences"[Title/Abstract] OR "gender equity"[MeSH Terms] OR "gender equity"[Text Word] OR "gender inequality"[Text Word] OR "gender discrimination"[Title/Abstract] OR "gender disparit*"[Title/Abstract]) AND ("terminal care"[MeSH Terms] OR "hospice care"[MeSH Terms] OR "end of life care"[Text Word] OR "palliative care"[MeSH Terms] OR "palliative care"[Text Word] OR "supportive care palliative"[Text Word] OR "palliative treatment"[Text Word] OR "palliative therapy"[Text Word] OR "palliative surgery"[Text Word])	91
2.	EMBASE	('gender differences':ti,ab,kw OR 'gender equity'/exp OR 'gender equity':ti,ab,kw OR 'gender inequality':ti,ab,kw OR 'gender discrimination':ti,ab,kw OR 'gender disparit*':ti,ab,kw) AND ('terminal care'/exp OR 'hospice care'/exp OR 'end of life care':ti,ab,kw OR 'palliative therapy'/exp OR 'palliative care':ti,ab,kw OR 'supportive care palliative':ti,ab,kw OR 'palliative treatment':ti,ab,kw OR 'palliative therapy':ti,ab,kw OR 'palliative surgery':ti,ab,kw)	170
3.	Scopus	(TITLE-ABS ("gender differences") OR INDEXTERMS ("gender equity") OR TITLE-ABS-KEY ("gender equity") OR TITLE-ABS-KEY ("gender inequality") OR TITLE-ABS ("gender discrimination") OR TITLE-ABS ("gender disparit*")) AND (INDEXTERMS ("terminal care") OR INDEXTERMS ("hospice care") OR TITLE-ABS-KEY ("end of life care") OR INDEXTERMS ("palliative care") OR TITLE-ABS-KEY ("palliative care") OR TITLE-ABS-KEY ("supportive care palliative") OR TITLE-ABS-KEY ("palliative treatment") OR TITLE-ABS-KEY ("palliative therapy") OR TITLE-ABS-KEY ("palliative surgery"))	136
4.	WOS	((TI="gender differences" OR AB="gender differences") OR ALL="gender equity" OR ALL="gender equity" OR ALL="gender inequality" OR (TI="gender discrimination" OR AB="gender discrimination") OR (TI="gender disparit*" OR AB="gender disparit*")) AND (ALL="terminal care" OR ALL="hospice care" OR ALL="end of life care" OR ALL="palliative care" OR ALL="palliative care" OR ALL="supportive care palliative" OR ALL="palliative treatment" OR ALL="palliative therapy" OR ALL="palliative surgery")	91
5.	Proquest	(TI,AB ("gender differences") OR MESH ("gender equity") OR TI,AB,IF ("gender equity") OR TI,AB,IF ("gender inequality") OR TI,AB ("gender discrimination") OR TI,AB (("gender disparities" OR "gender disparity"))) AND (MESH ("terminal care") OR MESH ("hospice care") OR TI,AB,IF ("end of life care") OR MESH ("palliative care") OR TI,AB,IF ("palliative care") OR TI,AB,IF ("supportive care palliative") OR TI,AB,IF ("palliative treatment") OR TI,AB,IF ("palliative therapy") OR TI,AB,IF ("palliative surgery"))	251
6.	CINAHL	((TI "gender differences" OR AB "gender differences") OR (MH "gender equity+") OR "gender equity" OR "gender inequality" OR (TI "gender discrimination" OR AB "gender discrimination") OR (TI "gender disparit*" OR AB "gender disparit*")) AND ((MH "terminal care+") OR (MH "hospice care+") OR "end of life care" OR (MH "palliative care+") OR "palliative care" OR "supportive care palliative" OR "palliative treatment" OR "palliative therapy" OR "palliative surgery")	59
